# Supplementary material for: Neural evidence that humans reuse strategies to solve new tasks
Source: PLoS Biol. 2025 Jun 5;23(6):e3003174. doi: 10.1371/journal.pbio.3003174 (PMC12140246; doi:10.1371/journal.pbio.3003174)
Supplement: Attachment — Submitted filename: Hall-McMaster_etal_Response_Letter.pdf [file pbio.3003174.s016.pdf]

Dear Reviewers,

Thank you for your time and attention to this manuscript, the positive feedback about our work, and the detailed and helpful comments on how to make it better. The revised version now details the similarities and differences between SF&GPI and alternative accounts (model-based processing with a partial model, configural learning and universal value function approximation). It includes new insights into the relationships between training and test choices, decoding evidence and test choices, and additional validation of the decoding procedure. We believe our manuscript has improved in its precision and robustness as a result of the review points raised. We have listed all review comments in a point-by-point manner below. Our replies and relevant excerpts from the text follow each comment. Moreover, all changes from the original documents are highlighted in red in the revised submission.

Thank you for reviewing our work,
Sam Hall-McMaster, Momchil Tomov, Samuel Gershman & Nicolas Schuck

Reviewer 1 (R1): "In the current paper, the authors investigate an interesting and timely question about the neural basis of a particular form of generalization, where human participants could re-use previous solutions in new problems. Behavior matched the predictions of their SF&GPI model, as previously behaviorally validated in an earlier publication. fMRI results focused on decoding representations of choice options, where the previously favored options were reactivated in new situations. Unexpectedly, the effect in the visual cortex was related to generalization behavior. The neural findings are interesting and novel. Overall, the paper was a pleasure to read. It has a very clear and comprehensive description of the experiment and results, and it is obvious that considerable care went into designing an interesting and well-controlled task and selecting appropriate methods. My concerns are primarily about interpretation, regarding a potential alternative account and the comparison to a model-based alternative."

Thank you for the generous and constructive comments on our manuscript.

R1.1: "The SF&GPI model is clear and it fits behavior well. However, I think it is important to describe how this model overlaps with or differs from an alternative perspective, based on configural / conjunctive learning, as highlighted by recent human imaging papers (e.g. Duncan et al. 2018 Neuron; Ballard et al. 2019 Nat Comm).

In the current task, one could construct an MF learning model that operates over configurations (cue screen displaying a +$ associated with a single gem), linking configurations to options (cities) based on reinforcement. This seems to be the same as a 'policy' in the SF&GPI model, but derived instead from an MF model based on conjunctions (and supported in implementation by the papers cited above).

For example, during training, consider the case where the cue screen configuration of [gem1 with +$ below] is followed by reward when city4 is chosen, while a cue screen configuration of [gem3 with +$ below] is followed by reward when city1 is chosen. Individual gems are not associated with reward, and individual cities are not associated with reward, but specifically the conjunction. Presenting that conjunction again will favor a response of the associated city, based on the stored values.

In the weather prediction task (e.g. Duncan et al.) this situation would be similar to a cue configuration of AB followed by reward when 'sun' is selected, while the configuration of AC or DB is followed by reward when 'rain' is selected.

In the current task, if these reinforced configuration-city links are carried over into the test trials, then the prediction is that participants would choose them instead of re-evaluating

the alternatives. The behavioral and neural predictions from this configural account seem like they would be very similar or exactly the same as those of the SF&GPI account. The authors should explain the overlap with the SF&GPI model, and whether and how a configural account could explain the current data.

Then a minor note about the neural predictions - in the configural account and also I think in the SF&GPI model, there is no extra need to activate the features (gem numbers), because the favored training policy can be selected without retrieval of these specific details. In a configural account, it also isn't clear that reactivation of the options would be expected to occur at all - especially if the configuration is well-learned. Perhaps this will not happen in the current experiment, as training is short and there are effectively many 'reversals' of configural responses across all the blocks."

Thank you for pointing this out. Configural learning is indeed a prominent account that has gained increasing neural support and it is interesting to consider whether this account might also explain the present findings. We have added the following material discussing this account to the revised manuscript.

Manuscript excerpt (Discussion, pg. 21):
Recent studies have also shown behavioural and neural evidence for reinforcement learning (RL) based on configurations of cues, over and above their individual elements (Duncan et al., 2018; Ballard et al., 2019). Applied to our setup, this would correspond to learning unique values for each (city, task) configuration. This account would not immediately capture the generalisation effects reported here. While an agent learning to respond to each configuration would be expected to learn optimal solutions to the training tasks, it would not have an obvious mechanism to map this knowledge to new tasks (i.e. new configurations of cues), the same issue facing a model free agent (Fig. S5). Nonetheless, configural accounts could in principle be extended to support generalisation to novel configurations using function approximation. This would allow an agent to smoothly interpolate between the values for different (city, task) configurations experienced during training, to compute approximate values for the new (city, task) configurations on test trials. Interestingly, this is precisely what UVFAs do. While our experiment was designed to disambiguate between SF&GPI and MB control using a complete model, we do see signatures of UVFA-like response patterns (Fig. S4B), even though our neural results appear to be inconsistent with UVFA predictions (Fig. S9B). Exploring UVFAs as a generalisation of configural RL in the multi-task domain could be an interesting direction for future investigation.

The reviewer's note about reactivation of the features is an important distinction between the configural learning and SF&GPI accounts. SF&GPI is explicitly constructed to solve new tasks through reactivating features expected under the policies acquired during learning.

<u>R1.2</u>: "I have two concerns about the strong interpretation of the support for the SF&GPI account of behavior over a model-based account. It seems that an MB agent with some reasonable additions - weighing noisy memory and trading of incentives versus costs of computation - would provide very similar results as the SF&GPI account, and so perhaps the interpretation can be tempered a bit.

First, subjects' actual task experiences during training, as shown in the individual subject data points shown in Figure 2C, only rarely include the two non-favored cities. Because they only have rare experience of the features (gem numbers) in those cities, and then even when experienced, they receive negative feedback, memory for them will be noisy and inaccurate, relative to the two other cities. The current MB agent assumes perfect memory for features in all cities, but this produces a bit of a straw model for comparison.

In an MB agent that takes into account the strength of memory, when gem value estimates are noisy and inaccurate, then when faced with a new situation in a test trial, this agent would weigh options by the degree of expected estimation errors, and, if the weight on memory in the decision were high enough, the MB agent would be biased to choose the previously optimal options - mirroring the SF&GPI agent.

The memory strength issues is something that the authors obliquely address in the supplement, to their credit. In the last block only, participants reported final gem count estimates and confidence ratings for each gem in each city. As this was limited to the last block, it likely reduces the power to detect some effects of memory errors and confidence and optimal policy. But nevertheless, the authors find a strong correlation between memory errors and the use of the SF&GPI policy, the same policy that would also be favored by an MB agent with uncertain memory. In the behavioral results, it would help to point to the correlation between error in gem count estimates for options disfavored during training & policy re-use.

Second, and less importantly, an MB agent with reasonable constraints may weigh whether full re-evaluation is worth the effort costs. In the current case, the relative reward improvement at test between the previously favored option and the new optimal option may have been insufficient to overcome the cost of doing a full MB evaluation (perhaps on top of the consideration of noisy memory).

Specifically, at test, it seems like the earnings benefit is only yielding improvements of at most 12.5% (or 10-20 points, as noted in the discussion section) over the option preferred during training. In contrast, during training, the earnings benefit over the next-best option seems to be at least a 200% improvement (though some alternatives are all negative). To

estimate the reward difference, of course, an MB agent would need to try this evaluation a few times in the first place, but once the minimal point gain in testing is checked in early blocks, the full evaluation could be dropped to save costs. (Adding this effort cost tradeoff involves some extra machinery than the simpler memory noise point.)

In the task description, I would suggest the authors add a note on the difference in the average delta between the optimal and next-best options during training and during test. (Based on quick calculations, that delta ranges from 60 to 180 points during training, but as described it is only 10-20 during test.) Of course, the authors were constrained in the experimental design by the arithmetic and conditions, so a greater benefit in test trials may not have been possible, but being clear about the values would help."

Taken together, these points about a more reasonable MB agent suggest that the current strong favoring of the SF&GPI agent be tempered a bit. These points do not affect the interpretation of the neural results."

Thank you for raising these excellent points. We agree that a model-based agent with noisy memory could behave in similar ways in our experiment to an SF&GPI agent, and that our conclusions should be appropriately tempered.

Following the reviewer's recommendations, we now draw attention to the correlation between the gem estimate error for the suboptimal training policies and policy reuse on test tasks, in the results section. This measure was only included at the very end of the experiment, as we did not want more regular assessment of the gem counts during scanning to influence participants' learning strategy.

Manuscript excerpt (Results, pg. 10):
The experiment also included an assessment of participants' explicit knowledge. Following scanning, participants estimated the number of gems that were present in each city during the final block of the experiment (Fig. S6). While we did not detect a difference in the mean gem estimate error across policies (corrected p-values>0.09, Fig. S6A), we observed a significant positive correlation between the estimate error for cities that were suboptimal during training and SF&GPI-consistent choices on test trials (Spearman's Rho=0.493, corrected p=0.016, Fig. S6B). This suggests a possible link between noisy memory for feature triplets associated with the suboptimal training policies and the reuse of successful past solutions.

We have also added total reward values for all task cue / feature triplet combinations, allowing readers to transparently view the difference in reward between the optimal policy and all other policies.

Manuscript excerpt (Results, pg. 6):
Reward for each combination of w and ϕ is presented in the methods (Fig. 4).

Manuscript excerpt (Method, pg. 30):
The reward for enacting each policy in each of the tasks is shown in Figure 4.

Manuscript excerpt (Method, pg. 31):

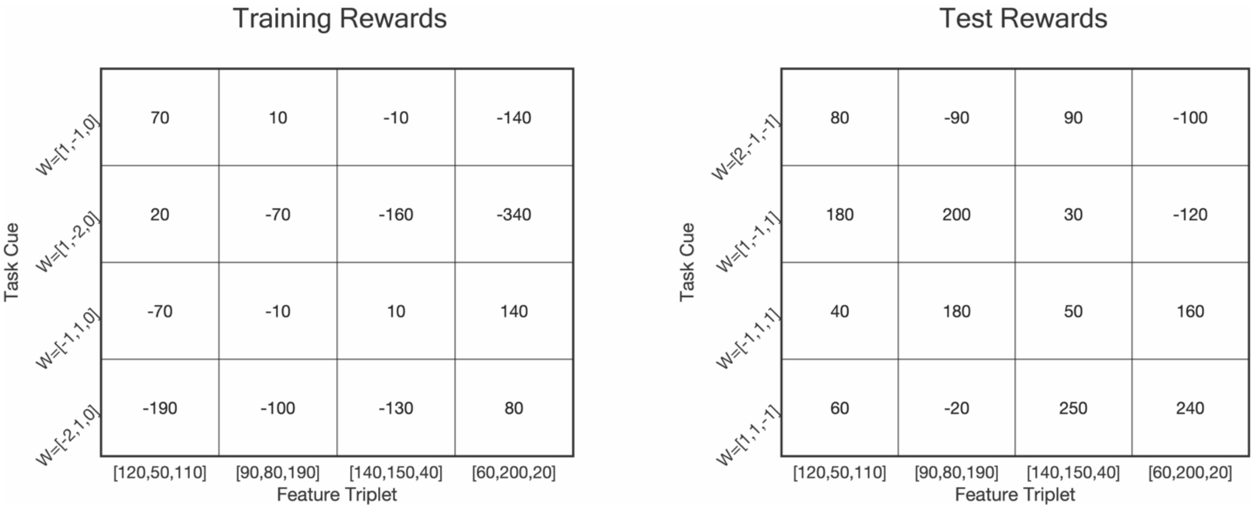

Figure 4. Reward outcomes for each combination of task cue and policy in the experiment. Rewards for training tasks are shown in the left-hand matrix. Rewards for test tasks are shown in the right-hand matrix. The order of feature triplets here is the same as other figures in the main text and supplement. [120,50,110] (or a permutation of these numbers) corresponds to 'Feature Triplet 1', [90,80,190] (or a permutation of these numbers) corresponds to 'Feature Triplet 2' and so on.

In addition, we now discuss that it is difficult to adjudicate between an SF&GPI agent and a modified MB agent with certain accommodations based on our results alone, and elaborate on how the reward-effort tradeoff in our experiment could influence the computational process used for transfer.

Manuscript excerpt (Discussion, pg. 24):
The most direct data we have on whether participants had a partial model in the current experiment were feature estimates collected after fMRI scanning. Specifically, participants were asked to estimate the features (gem numbers) for each city in the final experimental block (Fig. S6). If participants had a partial model of the environment, one prediction is that participants should have more accurate knowledge about features associated with the optimal training policies. We did not detect robust evidence for this prediction (corrected p-values>0.09, Fig. S6A). However, we did observe a significant correlation between having less accurate knowledge about features associated with the suboptimal training policies and the reuse of successful past solutions during test tasks (Spearman's Rho=0.493, p=0.016, Fig. S6B). One possibility based on this result is that participants with higher policy reuse had a partial model of the environment or noisier memory for certain features. Unfortunately, the present design can only arbitrate between a computational process based on SF&GPI and a MB process that uses a complete model of the environment. It is unable to arbitrate between SF&GPI and forms of MB processing that use a partial model of the environment or have noisy memory.

Manuscript excerpt (Discussion, pg. 25):
While we observed a high degree of reuse as predicted under an SF&GPI algorithm, it could be that the computational process would differ – with more equal evaluation across all options – if the reward prospect for previously unsuccessful policies had been higher at test. This would align with evidence that more computationally intensive forms of decision-making, such as MB planning, are adopted when the reward for accurate performance is heightened (Kool et al., 2017). The present experiment was not optimised to address meta-control between computational processes for different reward-effort trade-offs. Future research could therefore manipulate the difference in reward between successful and unsuccessful training policies during test tasks, to better understand the conditions under which an SF&GPI-like transfer process is (or is not) used.

Manuscript excerpt (Discussion, pg. 25):
Overall, the present study provides behavioural and neural evidence that generalisation to new tasks was more consistent with an SF&GPI-based algorithm than an MB algorithm using a full model of the environment. These results do not rule out an MB algorithm operating on a partial model of the environment, or with noisy memory for features associated with suboptimal training choices.

Finally, when referring to SF&GPI-consistent behaviour in the revised manuscript, we are careful to state that this is specifically in reference to an MB algorithm operating on a full model of the environment. We believe that this particular comparison is justified by the present data but agree with the reviewer that the present data do not rule out other forms of MB processing. As such, we have attempted to revise the manuscript to increase its precision and avoid giving the impression that our findings are inconsistent with all forms of MB control.

Manuscript excerpt (Abstract, pg. 2):
Neither model-free perseveration or model-based control using a complete model of the environment could explain choice behaviour.

Manuscript excerpt (Results, pg. 10):
Taken together, the results in this section indicate that participants were choosing among the optimal training policies on test trials in a reward-sensitive manner, a behavioural profile more consistent with the predictions of an SF&GPI algorithm than an MB algorithm operating on a full model of the environment.

Manuscript excerpt (Discussion, pg. 20):
Behavioural results showed that human choices on new tasks relied on reusing policies that were successful in previous tasks. While this strategy was less optimal than a model-based process using a full model of the environment, past policies were applied in a reward-sensitive manner that led to high performance … Our results speak towards a role of OTC and DLPFC in implementing an efficient form of generalisation that is superior to model-free behaviour, but less optimal than full model-based computation operating on a complete model of the environment.

R1.3: “ In the behavior-to-neural correlation analysis shown in Fig. 3F, it isn't clear how much this result may be associated with the response made on those trials. Controlling for the response made is an extra analysis done in the previous section, but it is omitted here. Please explain.”

Thank you for pointing this out. The challenge in controlling for the response in this case is that it forms part of our dependent measure. We would not necessarily expect neural evidence for SF&GPI taken from trials where a different policy was selected to correlate with SF&GPI-consistent behaviour, in line with what we see when we look at the brain behavior correlation on trials that were inconsistent with the SF&GPI model (OTC: Spearman’s Rho=0.186, uncorrected p=0.263, corrected p=0.526). We have therefore added additional correlation analyses examining the specificity of the neural predictor to the revised manuscript.

Manuscript excerpt (Results, pg. 18):
To examine whether neural coding in OTC and DLPFC had a functional connection to participant choices, we correlated average decoding strength for the more rewarding training policy (the orange dots in Fig. 3B) with the proportion of test trials in which participants generalised the more rewarding training policy. This revealed a significant positive correlation between neural activation of the more rewarding training policy in OTC and the implementation of that policy at test (Spearman's Rho=0.447, corrected p=0.010). The equivalent correlation was not detected for DLPFC (Spearman's Rho=0.115, corrected p=0.492). The correlation results held when using neural prioritisation towards the more rewarding training policy (the orange dots in Fig. 3C) as the neural variable (OTC: Spearman's Rho=0.433, corrected p=0.013; DLPFC: Spearman's Rho=0.161, corrected p=0.335, Fig. 3F). Neural coding in OTC was also directionally specific. Increased neural evidence for the more rewarding training policy in OTC showed a significant negative relation to the proportion of optimal MB choices at test (OTC: Spearman's Rho=-0.375, corrected p=0.041; DLPFC: Spearman's Rho=-0.098, corrected p=0.560).

<u>Minor points</u>
R1.4: "The number of subjects in the abstract isn't what went into the reported analysis, which seems to be 38."

Thank you for spotting this error. This has now been corrected.

Manuscript excerpt (Abstract, pg. 2):
Consistent with this idea, we found that human participants (n=38) learned optimal solutions to a set of training tasks and generalised them to novel test tasks in a reward selective manner.

R1.5: "For classifier training, the authors report the mean number of trials that go into training, but what is the minimum (and maximum) for each of these analyses?"

Minimum and maximum trial numbers for these analyses have now been added.

Manuscript excerpt (Results, policy decoding, pg. 12):
(M=53 trials per training class, SD=6.48, minimum=37, maximum=65).

Manuscript excerpt (Results, feature decoding, pg. 19):
(M=36 trials per training class, SD=2.44, minimum=29, maximum=39).

R1.6: "Did the associative reactivation of points on day 1 lead to significant classification of the point features, when tested on day 1 alone?"

Cross-validated tests using data from the associative memory paradigm alone indicated robust decoding evidence for point features in OTC, OFC, DLPFC but not MTL. We have added this information to the revised manuscript.

Manuscript excerpt (Results, pg. 19):
The training process was similar to the process used for policy decoding. One measurement volume (TR) per eligible trial was used as training input, taken 4-6s after the response screen onset. The time shift and smoothing used for each ROI were the same as those used to decode policies. This approach could successfully recover the 12 number targets when tested in held-out data from the associative memory paradigm in all ROIs except MTL (Fig. S11).

Manuscript excerpt (Supplement, pg. 12).

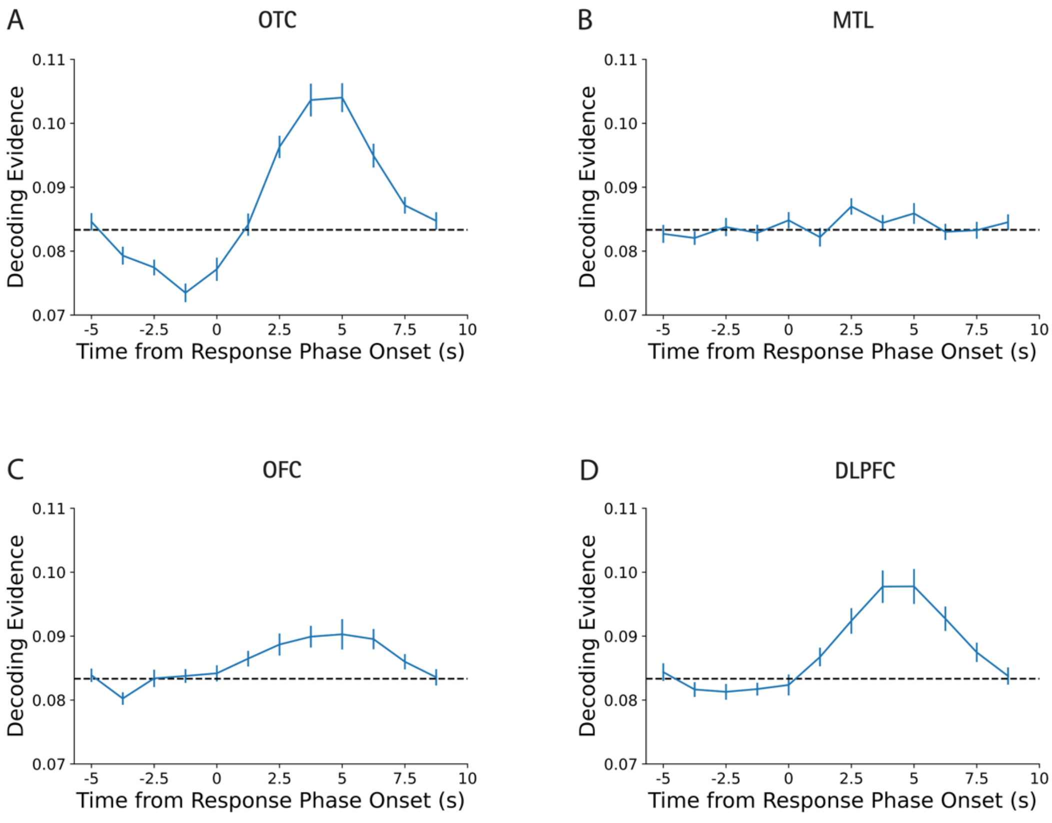

Figure S11. Validation tests of feature decoding during the associative memory paradigm. To test the validity of our feature decoding approach, we trained neural decoders to distinguish the target number presented during the response phase. Response phase onset was used for training to be as consistent as possible as the policy decoding approach in Figure S7, given the

associative memory paradigm did not have feedback after correct responses. The validation tests used the optimal smoothing and time shift for each ROI identified in Figure S7. Once the feature decoders were trained, we tested how well we could recover the target number on held out trials. One functional run was held out at a time to assess decoding performance. The process was repeated 25 times. Data were subsampled at random on each iteration to match the number of trials from each target class. A: Decoding time course for occipitotemporal cortex (OTC). Bars indicate standard error of the mean. The dotted line indicates chance based on 12 possible classes. B-D: The remaining panels follow the same structure as A but present validation results for different brain regions. These include the medial temporal lobe (MTL) in B, the orbitofrontal cortex (OFC) in C and the dorsolateral prefrontal cortex (DLPFC) in D. The target number could be recovered in OTC, OFC, DLPFC but not MTL using this approach.

R1.7: "Did the associative reactivation of cities on day 1 work on day 1 data, and did it lead to a similar-performing classifier the one built from day 2 data?"

The target cities could be recovered using neural data from all ROIs during the associative memory paradigm. However, we found that city decoding during the gem collector paradigm was higher in most ROIs when the decoders were trained using trials from that same paradigm. We have added these details to the revised manuscript.

Manuscript excerpt (Discussion, pg. 23):
Based on proposals that the MTL and OFC serve as predictive maps that encode information about future states (De Cothi & Barry, 2020; Geerts et al. 2020; Muhle-Karbe et al., 2023; Stachenfeld et al. 2017; Wimmer & Büchel, 2019), we predicted that features expected under the optimal training policies would be detected in these regions on test trials. We did not find evidence that this was the case. ... This could occur if feature numbers were represented with different neural patterns in the localiser task used to train the neural decoders than the main task. Cross-validated tests with policy decoders imply some support for this possibility, suggesting that decoders trained and tested on the main task tended to perform better, compared to those trained on the localiser and then tested on the main task (Fig. S13).

Manuscript excerpt (Supplement, pg. 14):

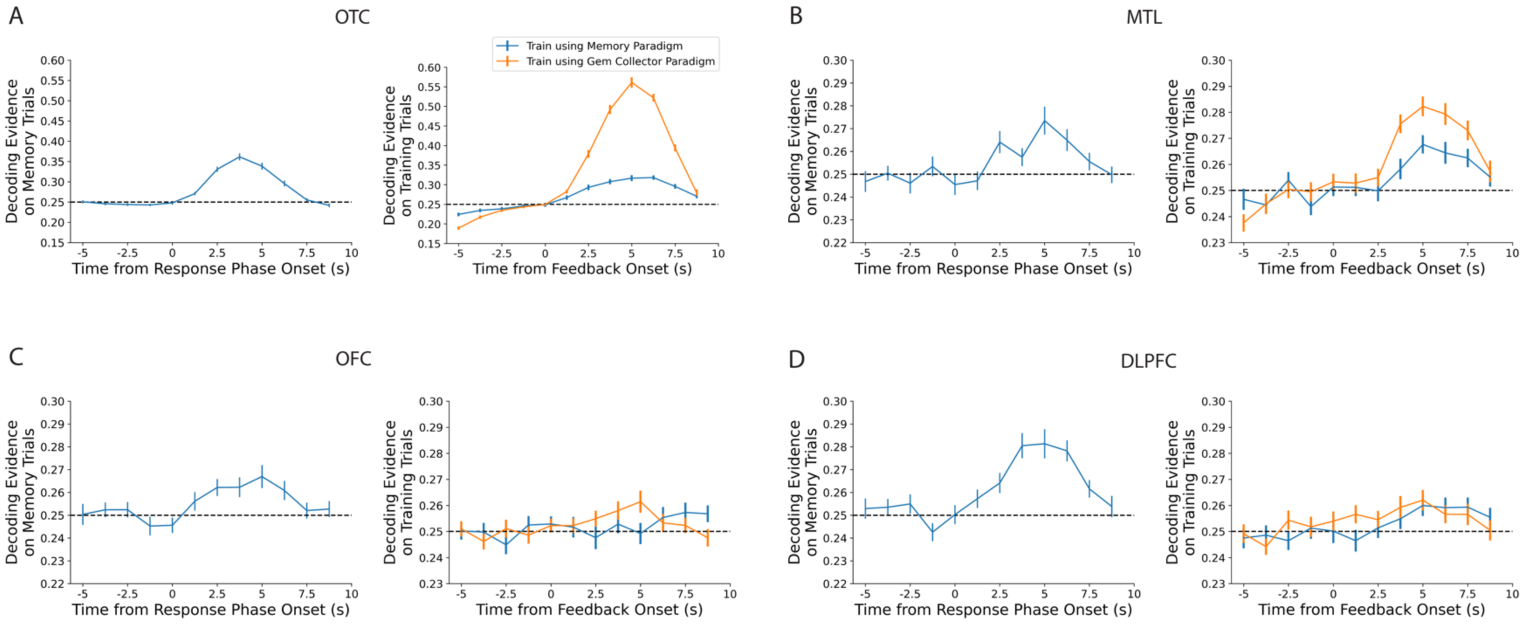

Figure S13. Comparison of city decoders trained on session one and session two data. Target cities could be recovered based on neural activity in all ROIs during the associative memory paradigm (session one). However, decoding evidence for cities during the gem collector paradigm (session two) was often higher when decoders were trained on the data from that same paradigm. A: Decoding evidence in OTC. The left subplot shows decoding evidence for target cities during the associative memory paradigm. The right subplot shows decoding evidence for cities selected on training trials of the gem collector paradigm, when the decoder was trained on data from session one (blue) or session two (orange). Bars indicate standard error of the mean and the dotted line indicates chance. B-D: Panels follow the same structure as A but present decoding time courses for different ROIs. A-D: Decoder training used the optimal smoothing and time shift for each ROI identified in Figure S7 and 25 subsampling iterations were performed to match the number of trials from each target class. Decoding evidence presented above is cross-validated, based on assessment on held-out test data.

R1.8: "Regarding the effects of feature triplet 4, was this related to the relative point difference between the best and next-best training option? I wasn't sure whether the features listed in order in the methods actually mapped to the order in the relevant supplemental figure, so it was hard to check."

This is an interesting point. To check whether reduced selection of cities associated with feature triplet 4 at test was due to a relative point difference between the two optimal training policies, we looked at the reward breakdown for each test task x feature triplet combination (shown in Fig. 4 below). We then computed the reward difference for the optimal training policies, leading

to feature triplet 1 ([120,50,110]) and feature triplet 4 ([60,200,20]). This resulted in the following absolute reward difference vector 180, 300, 120, 180. The specific test task in which participant responses were split between SF&GPI predicted choice and the MB predicted choice (resulting in reduced choices to feature triplet 4) had the cue [1,1,-1] (or a permutation of it), with an absolute reward difference of 180 points. This difference appears comparable to the reward difference on other test tasks, suggesting this was unlikely a systematic factor explaining reduced choices to triplet 4.

Manuscript excerpt (Method, pg. 31):

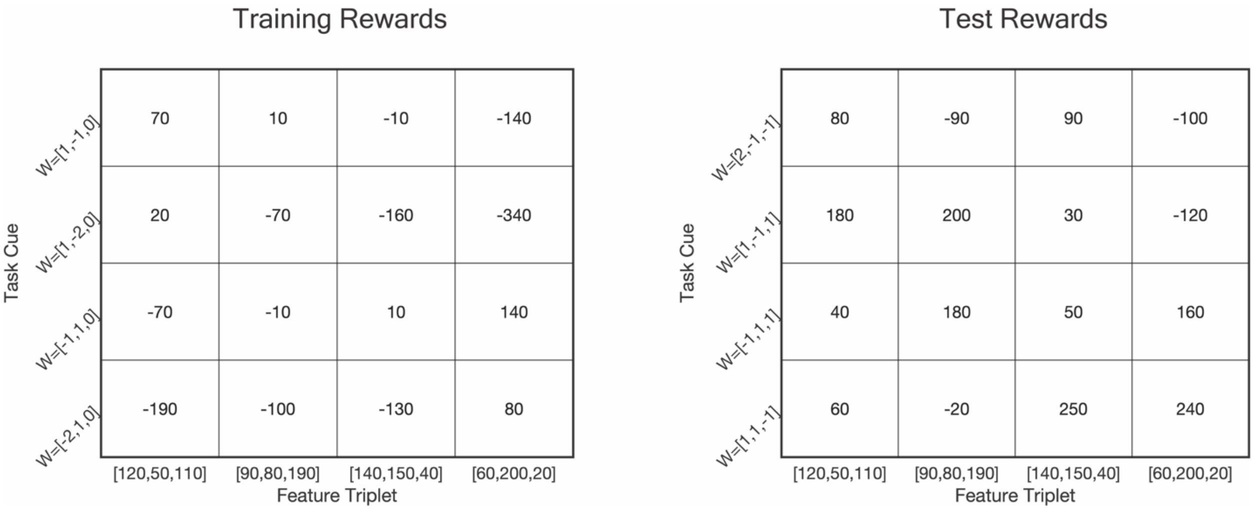

Figure 4. Reward outcomes for each combination of task cue and policy in the experiment. Rewards for training tasks are shown in the left-hand matrix. Rewards for test tasks are shown in the right-hand matrix. The order of feature triplets here is the same as other figures in the main text and supplement. [120,50,110] (or a permutation of these numbers) corresponds to 'Feature Triplet 1', [90,80,190] (or a permutation of these numbers) corresponds to 'Feature Triplet 2' and so on.

<u>Reviewer 2 (R2):</u>
"This study by Hall-McMaster and colleagues aims to test a few predictions about the neural substrates of generalization. Using a task called the gem collector task, the authors present behavioral evidence suggesting that when participants transferred prior knowledge of the environment to adapt to changing reward contingencies, their behavior aligned most closely with the successor features (SF)-generalized policy improvement (GPI) algorithm. By decoding fMRI data from several regions of interest (ROIs), they found that representations of optimal training policies remained stronger during test trials, even when those policies were no longer optimal. Furthermore, among the optimal training policies, the one that yielded higher rewards during test trials was more strongly activated.

Overall, the results are original and compelling, offering insights into the neural substrates of flexible learning in humans. The experimental design builds on a previous study by Tomov et al. (2021), and the data analyses consist of straightforward behavioral analyses and neural decoding. The statistical analyses appear sound and align with standard procedures in fMRI research (though we note that we are not experts in fMRI). The supplementary information and figures are helpful, and both the behavioral and neural data appear to be available online.

Although the findings are clearly presented, a deeper examination of both the behavioral and neural data could enhance their impact. Below, we present several questions and propose additional analyses that could further strengthen the study's claims and potentially uncover additional insights. Some of the proposed analyses are essential to fully support the current conclusions."

Thank you for the positive evaluation of our work and the constructive feedback on how to improve it.

R2.1: "The overall behavioral results are clear and convincing. However, there are a few subtle but important behavioral effects that could benefit from more explanation. First is that there are significant differences between the proportion of option 1 and option 4 choices, which is not predicted by any of the models. The discussion section mentioned that a Universal Value Function Approximator (UVFA) could explain this discrepancy. It would be informative to include predictions from that model to demonstrate that it cannot account for the dominant patterns observed in the behavior, and potentially in the neural data, as effectively as SF-GPI. Additionally, some subtleties in participants' behavior are discussed in the discussion section. These would make valuable supplementary analyses for the main findings and could be integrated into the results section for greater clarity and impact."

Thank you for raising these points. Based on the findings from Tomov et al. (2021), we optimised our experiment to distinguish between an SF&GPI algorithm and an MB algorithm that runs MB planning using a full model of the environment. Our main motivation was to test the neural predictions of SF&GPI. Although a UVFA-based process would not be expected for most participants a priori based on the findings from Tomov et al. (2021), we agree it is worth giving appropriate consideration.

The revised manuscript now includes theoretical predictions for UVFAs generated post hoc. These predictions are similar to human choice profiles. However, we found that the neural data were inconsistent with several UVFA predictions, which we detail in the revised version.

Manuscript excerpt (Supplement, pg. 4):

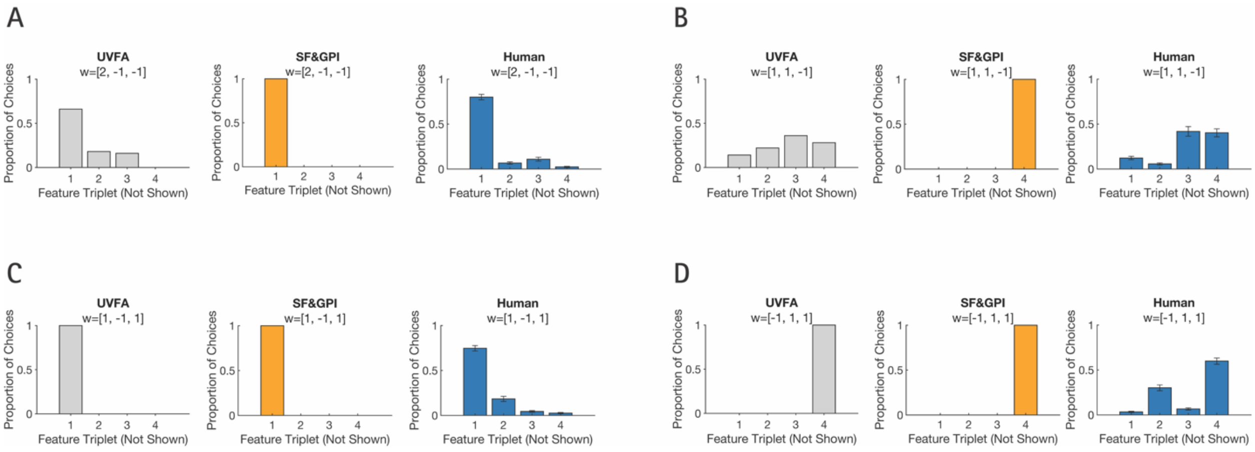

Figure S4. Predictions from a Universal Value Function Approximator (UVFA) algorithm. Each plot shows the proportion of choices (Y-axis) that lead to each feature triplet (X-axis). Each panel shows the choice profile for an individual test task. The cue for an individual task was a weight vector, w, which is indicated in the plot titles. These cues were called market values during the gem collector game. Each panel includes three plots: the theoretical predictions of a UVFA algorithm, the theoretical predictions of an SF&GPI algorithm and empirical choices from human participants. Bars indicate the standard error of the mean in plots with human data. UVFA predictions were generated post hoc using the implementation in Tomov et al. (2021).

Manuscript excerpt (Discussion, pg. 21):
Post hoc behavioural predictions of a UVFA process are included in Figure S4. These predictions show consistencies with human choice, suggesting that UVFA could offer an alternative account of behavior. However, the neural findings from this experiment appear to be inconsistent with several UVFA predictions. Vanilla UVFAs predict equal activation of all available actions to compute and compare Q-values. In contrast, OTC showed activation of the optimal training actions/policies at choice onset, followed by sustained activation of the more rewarding action/policy (Fig. 3D), whereas DLPFC showed activation of the more rewarding

action/policy (Fig. 3E). While this could be explained by a partial Markov decision process, in which people only evaluate actions that were reinforced during training, it would be inconsistent with the UVFA-like empirical choice profile on test task w=[1,1,-1] (Fig. S4B), in which a non-reinforced action is selected with high frequency. A UVFA explanation of the empirical choice profile on test task w=[1,1,-1] (Fig. S4B) would also predict approximately equal neural activation of both the objective best policy and the more rewarding training policy. Instead, we find that the more rewarding training policy is activated significantly more than the objective best policy, for which decoding evidence is at chance (Fig. S9B). Despite these neural inconsistencies, the behavioural similarities to UVFAs suggest that humans may utilise some kind of UVFA-like generalisation, perhaps in addition to SF&GPI-like policy reuse, in which the evaluated actions are not explicitly represented in the brain regions considered here. Exploring potential hybrid strategies that combine UVFAs with SF&GPI could be a promising area of future investigation.

Manuscript excerpt (Supplement, pg. 10):

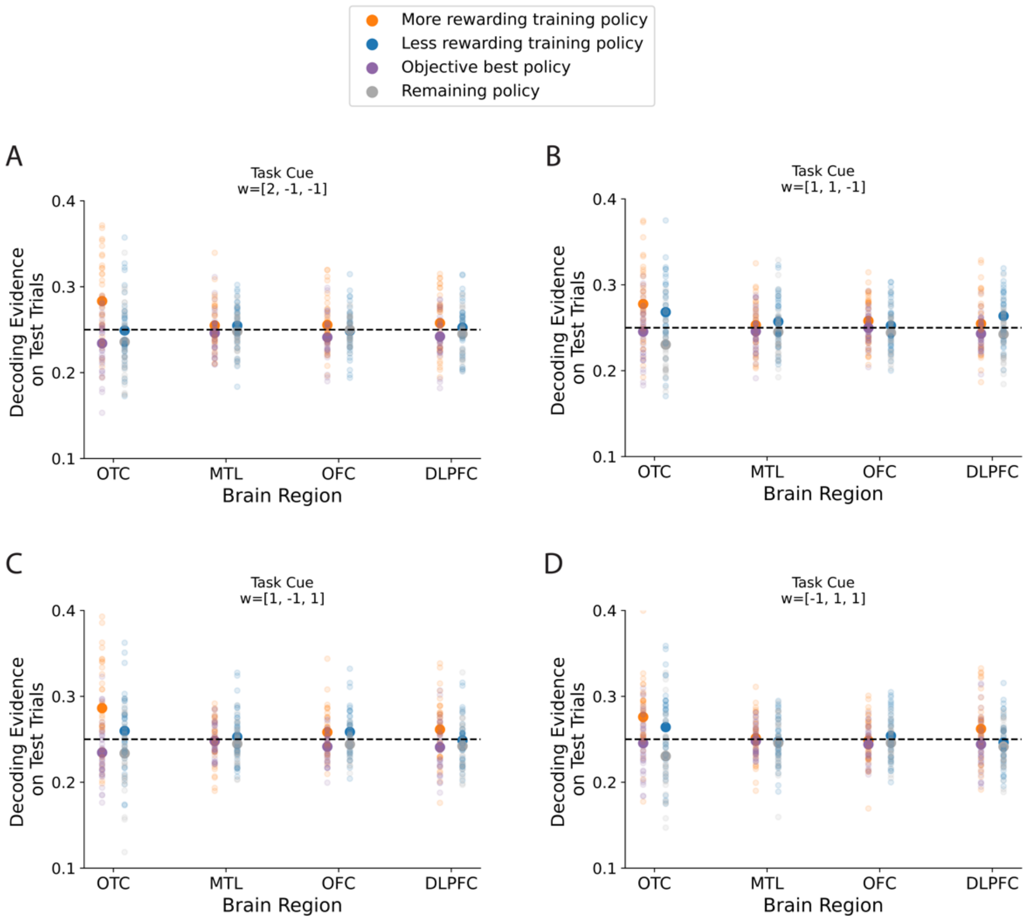

Figure S9. Decoding evidence for policies on each individual test task. Each panel shows average decoding evidence during a specific test task (y-axis) as a function of brain region (x-axis). Colours denote different policy categories and small translucent circles show data from individual participants. Panel B is especially relevant for testing whether the neural data are more consistent with SF&GPI or Universal Value Function Approximation (UVFA). The theoretical choice profiles for SF&GPI and UVFA are distinct for test task w=[1,1,-1] (Fig. S4). The theoretical choice profile for UVFA predicts: 1) that the more rewarding training policy and objective best policy will be activated on test task w=[1,1,-1] and 2) that the decoding evidence for these two policies will be comparable in magnitude. In contrast, we found that the more rewarding training policy was activated in OTC (t(37)=3.87, corrected p=0.005) but did not detect evidence that the objective best policy was activated (t(37)=-0.90, corrected p=0.750). Decoding evidence for the more rewarding training policy was also significantly higher than the objective best policy (t(37)=3.21, corrected p=0.014). Equivalent tests in DLPFC were not significant (t-values<1.66, corrected p-values>0.094). These follow up tests were restricted to OTC and DLPFC based on the decoding results in Fig. 3 of the main text. p-values were corrected for 6 tests using the Bonferroni-Holm correction (2 policy categories x 2 ROIs and 2 follow up tests assessing the difference in decoding evidence in each ROI).

To formalise the subtleties in human behaviour, our revised submission further includes statistical analyses for choices on individual test tasks.

Manuscript excerpt (Results, pg. 9):
The SF&GPI-predicted choice was made significantly more often than the alternatives on 3 of the 4 individual test tasks, with the remaining test task showing an even split between SF&GPI and MB choice (Fig. S3), an interesting behavioural signature of Universal Value Function Approximation (Fig. S4).

Manuscript excerpt (Supplement, pg. 3):
To formally examine human choice profiles on an individual task level, we compared whether participants made the SF&GPI predicted choice significantly more often than the alternatives. For example, using data from the test task w=[2,-1,-1] we compared whether the proportion of choices leading to feature triplet 1 was significantly higher than the proportions leading to feature triplet 2, 3 and 4. Repeating this process for each individual test task revealed that participants made the SF&GPI-predicted choice significantly more often in all cases except one (all t-values without the exception > 4.54, all corrected p-values without the exception < 0.001). The exception can be seen in panel B where the proportion of SF&GPI-predicted choices and MB-predicted choices were comparable ($M\phi(4)$=40.24% of test trials, $SD\phi(4)$=27.40, $M\phi(3)$=41.83%, $SD\phi(3)$=32.91, t(37)=-0.166, corrected p=0.869). Choice proportions were compared using paired samples t-tests and p-values were corrected for the 12 tests in this section using the Bonferroni-Holm correction.

R2.2: "Based on the choice proportions (Fig. 1C), it seems that participants did not use a strictly optimal policy during training, since the non-optimal options were chosen with some frequency. Did participants choose among the non-optimal options randomly (i.e., an epsilon-greedy policy) or did they favor any one of the non-optimal options in each task? Do individual differences in training trials predict their behavior in test trials (maybe explaining the subgroups in Fig. S2)? If so, this would provide even stronger evidence for transfer of training policies to test trials."

To investigate participants' selection of the non-optimal options, we compared the proportion of choices leading to the suboptimal feature triplets, $\phi(2)$ and $\phi(3)$, separately for each training task. After correction for four exploratory tests using the Bonferroni-Holm correction, we found no evidence for different choice frequencies among the suboptimal training policies in two training tasks (W=[1,-1,0]: $t(37)=0.72$, corrected $p=0.476$; W=[-1,1,0], $t(37)=-2.10$, corrected $p=0.086$), while for the two other training tasks choice proportions among the suboptimal training policies differed significantly (W=[1,-2,0]: $t(37)=3.44$, corrected $p=0.006$; W=[-2,1,0], $t(37)=-2.61$, corrected $p=0.039$). The cases where suboptimal option choice significantly differed appeared to have relatively small effect sizes, constituting a difference of approximately 2.25% of training trials (W=[1,-2,0]: difference in choice proportion between $\phi(2)$ and $\phi(3)$ = 0.026; W=[-2,1,0]: difference in choice proportion between $\phi(2)$ and $\phi(3)$ = -0.020). This suggests that the selection among non-optimal policies was not random for every task in the experiment but that preferences for one suboptimal training policy over another were numerically small.

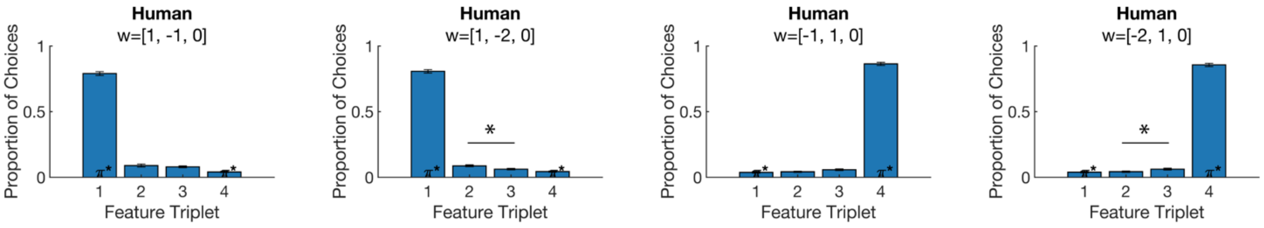

To assess whether individual differences in decisions during training trials were related to decisions on test trials, we tested the correlation between the proportion of optimal choices made during training and SF&GPI-consistent choices on test tasks. We have added the result of this correlation test to the revised manuscript.

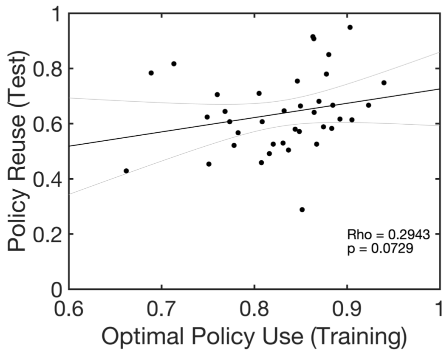

Manuscript excerpt (Results, pg. 9):
The proportion of SF&GPI-predicted choices on test tasks was positively related to the proportion of optimal choices during training but not to a significant degree (Spearman's Rho=0.294, corrected p=0.073).

To better understand the two subgroups in Fig. S2, we examined their training performance. Both groups had visually similar choice profiles on training tasks, selecting the optimal training policies at a much higher frequency than the suboptimal policies. Nonetheless, statistical tests indicated robust group differences in the extent of suboptimal choices. These statistical results are included in the revised submission.

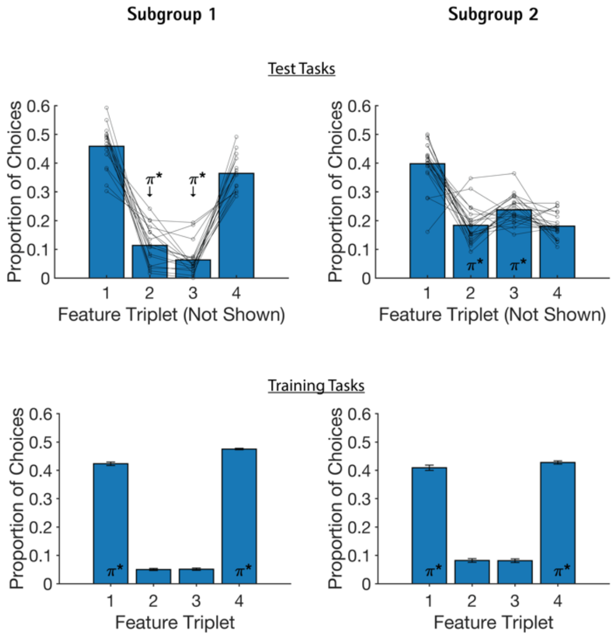

Manuscript excerpt (Results, pg. 9):
Participants could also be separated into subgroups based on how closely their behaviour matched the SF&GPI predictions (Fig. S2). The subgroup with less SF&GPI-consistent choices at test made significantly more choices leading to the suboptimal feature triplets $\phi(2)$ and $\phi(3)$ during training ($t(36)=4.88$, $p<0.001$).

Manuscript excerpt (Supplement, pg. 2):
An exploratory between-samples t-test comparing choices of the two subgroups on training trials showed that the subgroup with less SF&GPI-consistent choices at test (shown in C) made significantly more choices leading to the suboptimal feature triplets $\phi(2)$ and $\phi(3)$ during training (subgroup in panel B: $M_{\phi(2,3)}=5.07\%$ of training trials, $SD_{\phi(2,3)}=1.58$, subgroup in panel C: $M_{\phi(2,3)}=8.15\%$, $SD_{\phi(2,3)}=2.28$, $t(36)=4.88$, $p<0.001$).

R2.3: "For the fMRI results, we wonder why TRs after feedback onset were used for training the decoder, since it seems sensible that signal related to upcoming city choices could appear as early as cue onset. However, in Fig. S6, it looks like the representation of chosen city only arose after feedback onset. Does this suggest that during choice, the representation of city is different from during feedback? If so, would one expect different results if the TRs around cue onset were used for training?"

It is indeed plausible that activation related to the upcoming city choice could occur following task cue onset. The original validation analyses locked to feedback onset do not necessarily imply that cities were not activated following cue onset, as the timing of feedback onset is jittered relative to cue onset (Figure 1). We focused on the feedback period because additional validation analyses that locked decoder training and assessment to task cue presentation indicated that recovering the cities was more effective when training data was based on feedback presentation. We have added these validation tests to the revised submission.

Manuscript excerpt (Results, pg. 12):
The specific time lag used for each ROI was determined in validation analyses of the training tasks, independent from our predictions about test task activity (see Fig. S7). Validation analyses further showed that decoder training locked to feedback onset on training tasks was more effective than decoder training locked to task cue onset (see Fig. S8).

Manuscript excerpt (Supplement, pg. 9):

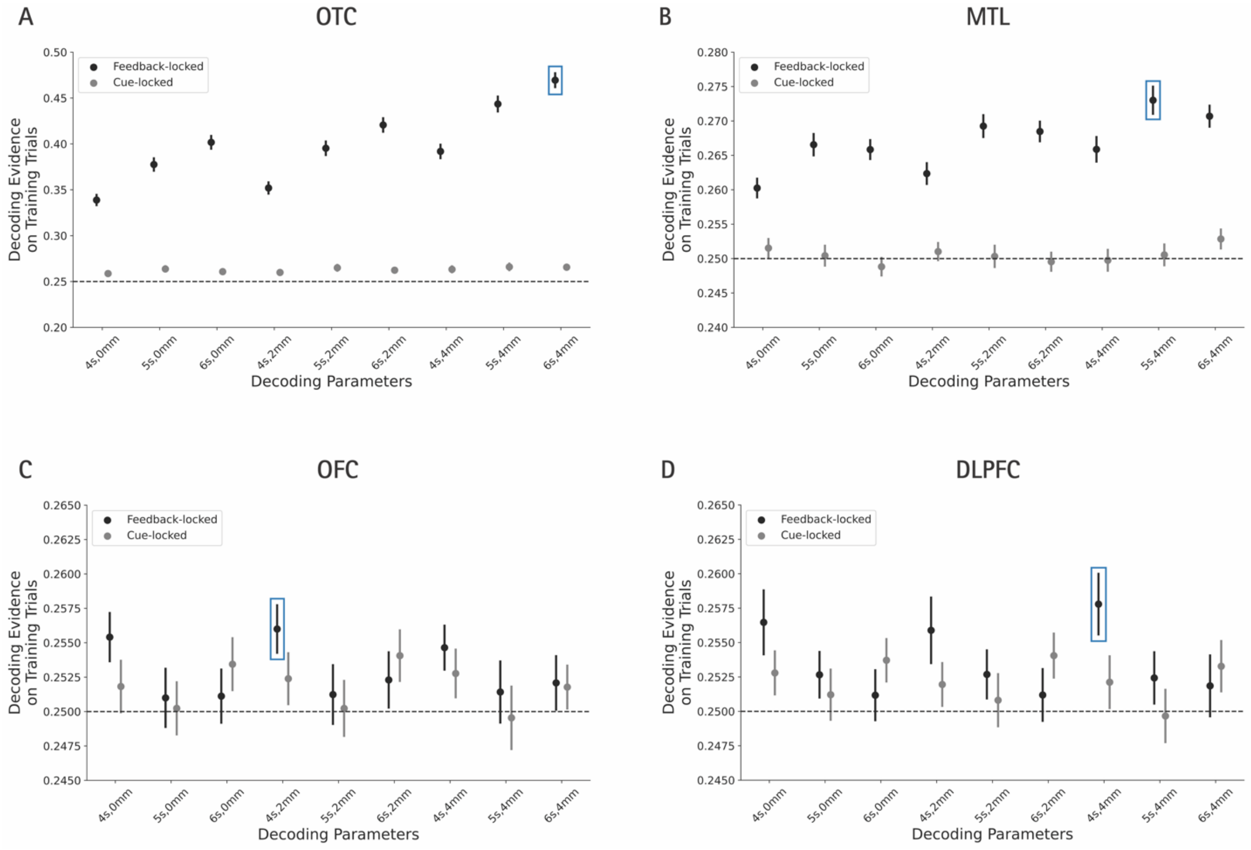

Figure S8. Comparison of cue-locked and feedback-locked decoding. It is possible that the activation of city stimuli could occur directly following task cue onset. We therefore performed additional validation tests, examining the decoding evidence on training tasks when decoder training was locked to task cue onset, rather than feedback onset. Decoding based on feedback onset was found to be more effective in all ROIs. Panels A-D were generated using the same approach described in Figure S7. For cue-locked decoding, the decoder was trained using data with a time shift of +4s, +5s, or +6s from cue onset and evaluated from cue onset on held-out trials. Decoding evidence above represents evidence for the city selected on each training trial, averaged from 2.5-8.75s following the onset of the relevant trial event (cue presentation or feedback presentation). A: Validation results for occipitotemporal cortex (OTC). Bars indicate standard error of the mean. The blue box denotes the decoding parameters with the highest validation performance. B-D: The remaining panels follow the same structure as A but present validation results for different brain regions. These include the medial temporal lobe (MTL) in B, the orbitofrontal cortex (OFC) in C and the dorsolateral prefrontal cortex (DLPFC) in D.

R2.4: "It is surprising that there is such a strong and robust effect in occipitotemporal cortex (OTC) but not frontal regions for this value-based task. In general, what is the implication for this result for the neural substrates/mechanisms of reinforcement learning? Where does the value information that modulates OTC activity come from, if not in OFC, DLPFC, or MTL? Could these regions represent some other task-related variables (maybe related to motor response)?"

We agree that the strong and robust effects in OTC are surprising. Nonetheless, there is emerging evidence that OTC might be involved in sophisticated predictions about future states. Moreover, it is plausible that other brain regions could have encoded other variables (such as value) and provided input to OTC. In fMRI, the signal to noise ratio also tends to be lower in frontal and medial-temporal regions (Weiskopf et al., 2006), reinforcing the notion that the absence of effects in OFC and MTL should be interpreted with caution. We have added these considerations to the revised manuscript.

Manuscript excerpt (Discussion, pg. 21):
While OTC is not a usual candidate for experiments on RL and decision-making, we note recent reports that neural replay decoded from OTC relates to the formation of successor representations (Wittkuhn et al., 2024), which are one of the theoretical foundations of SF&GPI.

Manuscript excerpt (Discussion, pg. 23):
It is also possible that OFC and MTL were engaged during the experiment but encoded other variables. For instance, one influential view of OFC function is that the OFC represents the prospect of reward for different options during decision-making (Kahnt et al. 2010, Knudsen & Wallis, 2022). The present experiment was not optimised to decode value signals due to the wide variation and largely unsystematic distribution of the rewards across training and test tasks (Fig. 4), a compromise that arose from the other factors we needed to balance in the experiment to test our central predictions. Some studies also suggest that hippocampal signalling may modulate the reinstatement strength of neural patterns in visual areas (Bosch et al., 2014; Hindy et al. 2016). Based on these factors, one speculative possibility could be that value information in OFC and hippocampal signalling modulate OTC and DLPFC activity on new tasks, in favour of successful past solutions.

R2.5: "Since behavioral results suggest that participants are not simply performing SF&GPI, we wonder if the lack of activation and prioritization of optimal training policies in OFC and MTL reflect a true lack of encoding, or that these areas encode some other type of decision signals (like more model-based signals), so that on average no policy is favored more than other ones. Maybe looking at the decoding evidence on a task-by-task basis will resolve this."

Thank you for this interesting suggestion. In the case of an MB algorithm operating on a full environmental model, the prediction indeed is that the agent would simulate expected rewards for all four policies. However, assuming the agent then represents the optimal policy for each test task, the average decoding evidence would not be distributed evenly across the four policies. This is because the test tasks were designed so that the optimal policies on test trials led to feature triplets $\phi(2)$ and $\phi(3)$ but never $\phi(1)$ and $\phi(4)$. Based on this, a potential signature of an MB process (using a full environmental model) would be significant decoding evidence of policies that lead to $\phi(2)$ and $\phi(3)$. When we first look at the decoding evidence averaged over all tasks in this area, our data suggest this was not the case.

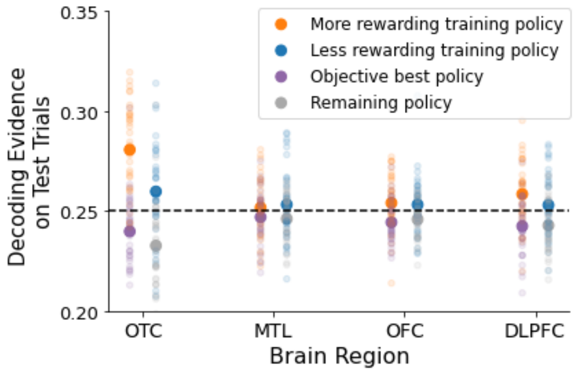

When we visualise the decoding evidence on a test-by-task basis, we neither see activation of the model-based policy in MTL or OFC during the four test tasks. We have added decoding evidence on each task to the supplemental materials.

Manuscript excerpt (Results, pg. 12):
Decoding evidence on individual test tasks is included in the supplement (Fig. S9).

Manuscript excerpt (Supplement, pg. 10):

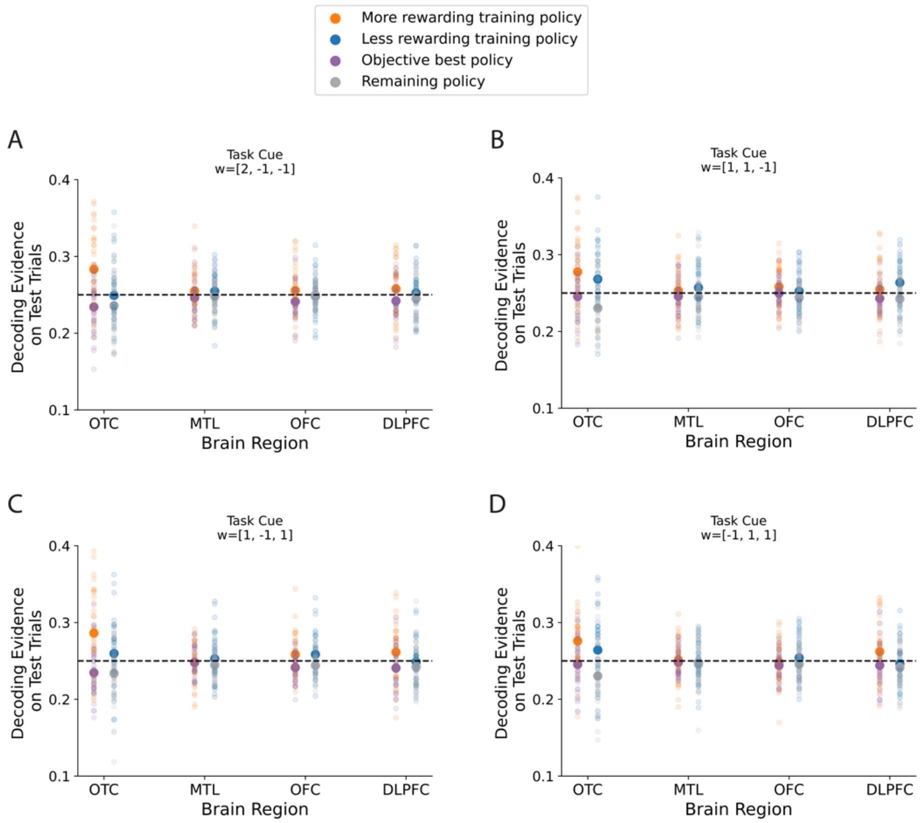

Figure S9. Decoding evidence for policies on each individual test task. Each panel shows average decoding evidence during a specific test task (y-axis) as a function of brain region (x-axis). Colours denote different policy categories and small translucent circles show data from individual participants. ...

<u>Minor point:</u>

R2.6: "The caption for Fig. 3F is incorrectly labeled as Fig. 3D. Additionally, it would be helpful to label each panel in this figure individually for clarity."

Thank you for spotting this oversight. We have corrected the caption label in the revised text and labelled each panel in the figure individually for clarity.
