# Supplementary material for: Neural evidence that humans reuse strategies to solve new tasks
Source: PLoS Biol. 2025 Jun 5;23(6):e3003174. doi: 10.1371/journal.pbio.3003174 (PMC12140246; doi:10.1371/journal.pbio.3003174)
Supplement: Attachment — Submitted filename: Hall-McMaster_etal_response_to_reviewers.docx [file pbio.3003174.s017.docx]

Reviewer points:

Reviewer #1 (G. Elliott Wimmer): **“Thank you for your consideration of a model-based alternative with noisy memory; I believe those adjustments have significantly improved the paper.**

**Thank you also for elaborating on the SF&GPI model relative to a configural learning account.  I miss-estimated what would be needed for test phase performance in a configural account, which would indeed involve some kind of generalization or additional combination of features during training, e.g., for the 4th test task [1, 1, -1], as listed in the methods.**

**Otherwise, a simple (but not at all formalized!) 'pick the highest combination that has been previously reinforced' kind of generalization would appear to solve 3 of 4 tests, given that three have a positive gem value that aligns with training (where there was only 1 positive value in the sets).**

**As an aside, it is interesting that the behavior for test [1, 1, -1], which has the only mismatch with a (not formalized) configural learning + generalization account, shows the lowest level of behavior consistent with the SF&GPI account. If a configural + generalization strategy was dominating in general, in this case, the lack of match could prompt the use of additional evaluation for this specific test case.”**

Thank you for your excellent insights and comments throughout peer review. Indeed, it is interesting to consider a heuristic strategy that focuses on the most rewarded feature of each test task, and looks for the alignment of that feature with training tasks. We have added the following material discussing this to the revised submission.

Manuscript excerpt (Discussion, pg. 22):

*A final account of generalisation behaviour could be a heuristic strategy, in which participants focus exclusively on the feature with the highest positive reward weight. Participants could then retrieve a policy from the training tasks that shared this top ranked feature. Such a heuristic would lead to SF&GPI-like behaviour in three test cases. However, it would not predict neural activation of the less rewarding training policy, as there would be no need to consider other features or policies once the top-ranked gem had been identified. Therefore, this heuristic strategy would not explain the neural effects in OTC, where both the more and less rewarding training policies were activated on test tasks (Fig. 3C-D).*

Reviewer #2 (Alireza Soltani): **“The authors have adequately addressed all of our concerns. We have no further comments.”**

Thank you very much for the constructive and helpful comments during peer review.
